# Intersection of Stargardt Dystrophy and AIDS: A Case Report

**DOI:** 10.7759/cureus.77828

**Published:** 2025-01-22

**Authors:** Taha Boutaj, Hamza Lazaar, Abdellah Amazouzi, Samira Tachfouti, Lalla Ouafa Cherkaoui

**Affiliations:** 1 Ophthalmology A, Hopital des Specialités, Centre Hospitalier Universitaire (CHU) Ibn Sina, Rabat, MAR; 2 Medicine, Hopital des Specialités, Centre Hospitalier Universitaire (CHU) Ibn Sina, Rabat, MAR; 3 Ophthalmology A, Hopital des Specialités, Mohammed V University, Rabat, MAR

**Keywords:** hiv aids, infection, retina, retinal dystrophy, stargardt disease

## Abstract

The co-occurrence of Stargardt disease, hereditary macular dystrophy, and advanced AIDS is a very rare association. The intersection of these two conditions raises important clinical considerations, particularly in differentiating retinal changes induced by macular dystrophy from those associated with human immunodeficiency virus (HIV) infection or antiretroviral therapy (ART). In this case, a 54-year-old woman with a history of advanced HIV presented with severe visual impairment, with finger-counting acuity in both eyes. Ophthalmic examination revealed extensive bilateral retinal atrophy involving the posterior pole and mid-peripheral retina, along with macular pigmentary migration lesions. Imaging studies, including autofluorescence and fluorescein angiography, demonstrated a centrally hyperfluorescent area and a large hypofluorescent zone with surrounding hyperfluorescence. Optical coherence tomography (OCT) confirmed abnormal hyper-reflectivity in the outer retinal layers and an enlarged foveal depression. Genetic testing confirmed two biallelic pathogenic variants in the ABCA4 gene, validating the diagnosis of Stargardt disease. The patient was also tested for systemic infections, such as syphilis and toxoplasmosis, to rule out opportunistic ocular infections associated with HIV. Her management was tailored to address both her retinal dystrophy and her immunosuppressive state. This case underscores the need for regular ophthalmological monitoring in patients with hereditary retinal dystrophies and HIV, particularly those on ART. Further research is warranted to explore the impact of HIV and its treatments on the progression of macular dystrophies like Stargardt disease.

## Introduction

Stargardt disease is a hereditary macular dystrophy and one of the most common causes of vision loss in both children and adults [1]. It is characterized by progressive degeneration of the retina, often leading to significant visual impairment. The disease is linked to mutations in the ABCA4 gene, following an autosomal recessive inheritance pattern [2].

Human immunodeficiency virus (HIV) is a virus that attacks the immune system. Without treatment, it can lead to acquired immunodeficiency syndrome (AIDS), the most advanced stage of HIV infection, where the body’s ability to fight infections is severely compromised. While much is known about the genetic underpinnings and clinical presentation of Stargardt disease, its interaction with systemic conditions like HIV/AIDS remains largely unexplored.

This case highlights the rare intersection of Stargardt disease and advanced HIV in a 54-year-old woman. Severe immunosuppression due to AIDS may influence the progression of hereditary retinal conditions, potentially exacerbating retinal degeneration. The case underscores the need for thorough ophthalmological evaluation in patients with coexisting systemic and hereditary conditions, as well as the importance of interdisciplinary collaboration in their management. By documenting this case, we aim to contribute to the understanding of how systemic diseases like HIV/AIDS can impact the course of retinal dystrophies, emphasizing the necessity of vigilant monitoring and tailored therapeutic approaches.

## Case presentation

We report the case of a 54-year-old female patient diagnosed with advanced AIDS, who was referred by her internist for an ophthalmological assessment. The patient was diagnosed with HIV 15 years ago. She was initially diagnosed with HIV following recurrent respiratory infections and an unintentional weight loss of 12 kg over six months. The diagnosis was confirmed via a positive HIV antibody test, followed by a Western blot confirmation and a CD4 count of 400 cells/mm³ at the time of diagnosis. Antiretroviral therapy (ART) was initiated shortly after confirmation of the diagnosis. Her condition has since progressed to AIDS, with a recent CD4 count of 80 cells/mm³. She was on ART, including tenofovir, emtricitabine, and efavirenz. There was no history of hospital admission to an intensive care unit (ICU), but she had been treated for recurrent pneumocystis pneumonia. During the interview, the patient reported a subtle decrease in visual acuity since childhood, for which she had never sought consultation.

**Figure 1:**
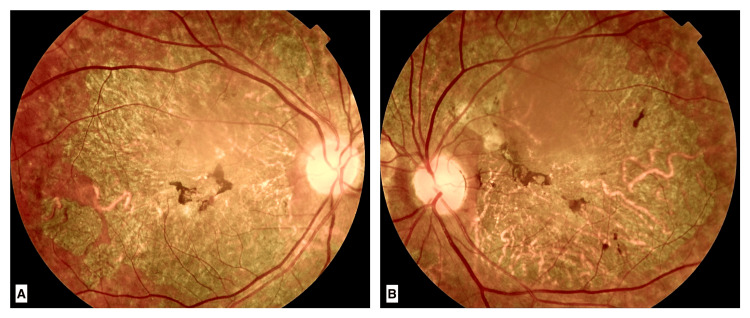
Fundus photographs of the right eye (A) and left eye (B) A large area of retinal atrophy is seen affecting the posterior pole and mid-peripheral retina, exposing the choroid. Pigment migration lesions are noted in the macular region.

On examination, visual acuity was limited to counting fingers at 3 meters in both eyes, with no improvement possible through optical correction. Anterior segment examination with a slit lamp revealed early nuclear cataracts in both eyes, with no signs of vitreous inflammation. Fundus examination showed marked bilateral retinal atrophy involving both the posterior pole and mid-peripheral retina, with choroidal exposure. The loss of the foveal reflex, along with pigment migration lesions in the macular region, was suggestive of a hereditary retinal dystrophy (Figure 1).

**Figure 2:**
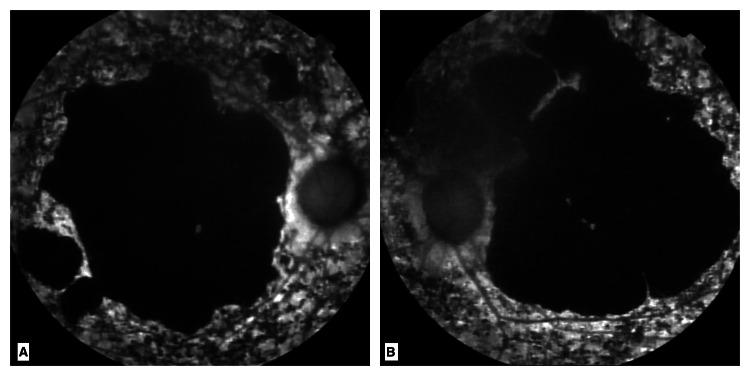
Autofluorescence images of the right eye (A) and left eye (B) Total macular atrophy is noted; a large central area that is completely hypoautofluorescent is visualized.

Autofluorescence imaging confirmed macular atrophy with a centrally hypoautofluorescent area indicative of advanced retinal atrophy (Figure 2).

**Figure 3:**
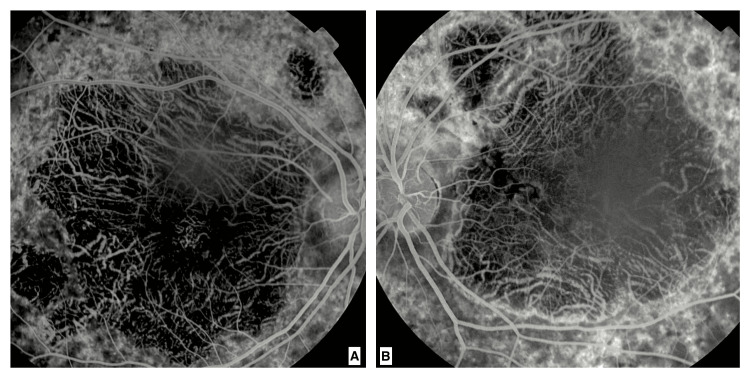
Early-phase fluorescein angiography of the right eye (A) and left eye (B) A large hypofluorescent area in the macular region between the separation of the superior and inferior temporal arcades, surrounded by a hyperfluorescent rim with abnormal visualization of choroidal vessel filling, is noted.

Fluorescein angiography revealed a large hypofluorescent zone in the macular region, bordered by a hyperfluorescent rim, with abnormal visualization of the choroidal vessels. These findings were consistent with a macular dystrophy of the Stargardt type (Figure 3).

**Figure 4:**
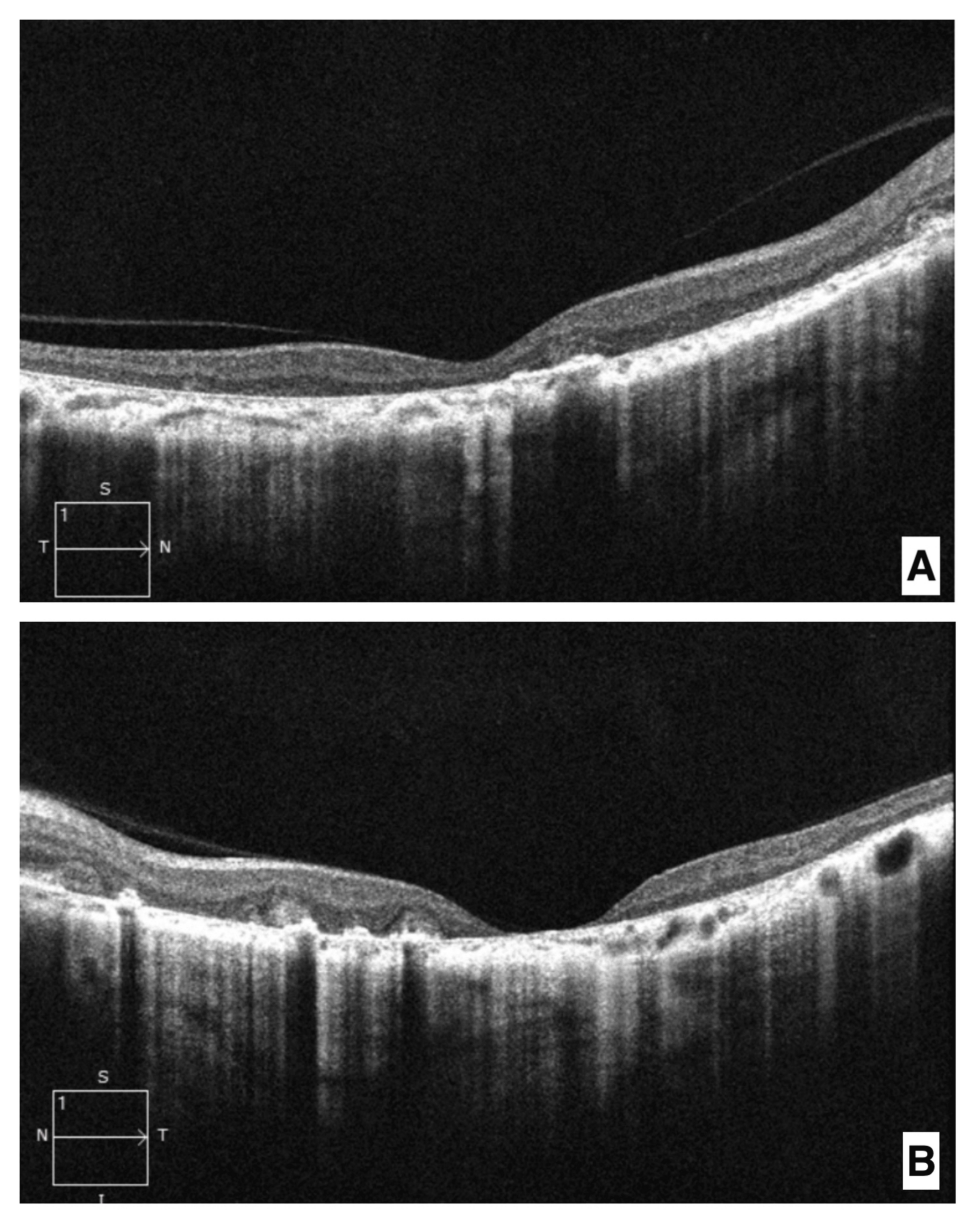
Macular optical coherence tomography (OCT) of the right eye (A) and left eye (B) Abnormal hyperreflectivity of the outer retinal layers with posterior shadowing, indicative of macular atrophy and widening of the foveal depression is noted.

Optical coherence tomography (OCT) showed abnormal hyper-reflectivity of the outer retinal layers, associated with an enlarged foveal depression, indicative of significant loss of central retinal structure (Figure 4).

Electroretinography (ERG) was performed, revealing a significant reduction in both scotopic and photopic responses, consistent with generalized retinal dysfunction.

Further tests for syphilis and toxoplasmosis were negative, ruling out these infections as potential contributors to the retinal damage observed.

Given the bilateral and symmetrical nature of the retinal involvement, along with the chronic and insidious visual acuity loss, the diagnosis of hereditary retinal dystrophy progressing towards atrophy was strongly suspected. Genetic testing was performed using next-generation sequencing (NGS), focusing on a large gene panel that includes genes associated with macular dystrophies. The testing revealed two biallelic pathogenic variants in the ABCA4 gene, consistent with the diagnosis of Stargardt disease.

The first variant, classified as "likely pathogenic" according to the American College of Medical Genetics and Genomics (ACMG) and the Association for Molecular Pathology (AMP) guidelines, was a missense variant: c.2588G>C in exon 13. This variant leads to an amino acid substitution (p.Gly863Ala), which has been previously reported in patients with Stargardt disease and is supported by in silico predictions (PolyPhen-2, SIFT) suggesting a damaging effect on protein function [3].

The second variant, classified as "pathogenic", was identified as a frameshift mutation: c.5461-10T>C in intron 39. This variant is known to lead to aberrant splicing, resulting in a truncated ABCA4 protein. Sanger sequencing was used to confirm both variants. This genetic combination confirms the diagnosis of autosomal recessive Stargardt disease.

A detailed literature review (GnomAD, ClinVar) indicated that these variants have been reported in affected individuals but are absent or extremely rare in large control databases, further supporting their pathogenicity [4]. No other significant variants were found in the panel, and no family members were available for segregation analysis; the genetic findings conclusively confirm Stargardt disease in this patient.

These clinical, electrophysiological, and genetic findings, combined with negative results for other infections, confirm with certainty the diagnosis of Stargardt macular dystrophy in the context of advanced HIV.

## Discussion

Stargardt disease is a prevalent hereditary cause of irreversible vision loss, affecting both children and adults. Advances in imaging technologies and genetic sequencing have enabled earlier and more precise diagnosis of this condition [1]. The disease follows an autosomal recessive inheritance pattern and is linked to mutations in the ABCA4 gene [2].

Currently, no similar cases have been reported in the literature; no studies have established a connection between HIV and hereditary retinal dystrophy. The association of Stargardt macular dystrophy with secondary viral retinitis due to HIV is exceptional.

Human immunodeficiency virus causes modifications in the outer retinal layers, particularly in the retinal pigment epithelium and the photoreceptor outer segments, leading to more significant progression towards retinal atrophy [5]. This could explain the severity of retinal damage in our case, where immunosuppression compounded the preexisting hereditary dystrophy. The role of immunosuppression in exacerbating retinal dystrophies is not well understood, but it is plausible that reduced immune defenses may worsen ongoing degenerative processes, particularly in the context of a genetic predisposition like the ABCA4 mutation [6].

Three hypotheses are worth discussing: chronic inflammation, exposure to opportunistic infections, and the side effects of antiretroviral treatments may all play a role in worsening retinal atrophy.

Chronic inflammation

Even when well-controlled by treatment, HIV is often associated with systemic inflammation [7]. This inflammation can affect retinal cells, particularly the photoreceptors and the retinal pigment epithelium, contributing to the progression of retinal atrophy. Moreover, patients with hereditary retinal dystrophies like Stargardt disease exhibit increased oxidative stress in the retina. Thus, HIV, combined with antiretroviral treatment, could exacerbate this oxidative stress, leading to more rapid retinal degeneration [8].

Opportunistic infections

Studies on retinal dystrophies in patients with HIV are almost non-existent but reported cases of other retinal conditions, such as cytomegalovirus retinitis, suggest that immunosuppression can accelerate retinal tissue degeneration [9]. This infection can cause severe necrotizing retinitis, leading to rapid vision loss. In the context of Stargardt dystrophy, such an infection could hasten the degeneration of already compromised retinal tissues.

Antiretroviral treatments

While effective in controlling HIV, antiretroviral treatments are associated with various ocular side effects; they can potentially contribute to systemic oxidative stress and inflammation, both of which are known to play roles in retinal degeneration [10]. Indinavir can enhance retinal dehydrogenase activity, leading to an increased generation of reactive oxygen species. This may result in elevated oxidative stress within the neuroretina [11]. Although these effects are rarely specifically associated with Stargardt disease, prolonged use of antiretroviral treatments could exacerbate retinal degeneration in patients with an underlying genetic mutation. The ABCA4 mutation responsible for Stargardt disease leads to the accumulation of toxic lipofuscin in the retinal pigment epithelium [12]. Human immunodeficiency virus-related immunosuppression could interfere with these mechanisms.

Although Stargardt disease was the most likely diagnosis given the clinical presentation, other macular dystrophies were considered: The ERG results, demonstrating reduced scotopic and photopic responses, are crucial in supporting the diagnosis of Stargardt disease, as they point to widespread retinal dysfunction beyond the macula. An ERG can be an important tool in differentiating between macular dystrophies like Stargardt disease and cone-rod dystrophies, where more significant photopic abnormalities might be expected [13]. In our case, the generalized retinal dysfunction detected by ERG is consistent with the diagnosis of Stargardt disease and helped exclude other hereditary or systemic causes of retinal degeneration.

PRPH2 gene mutations, which can cause pattern dystrophy, were also considered, as these mutations can present with similar retinal findings, including pigment migration and macular atrophy. However, genetic testing did not reveal PRPH2 variants, further supporting the diagnosis of Stargardt disease. Other causes of macular degeneration, such as medication toxicity, were ruled out given the patient’s stable long-term ART regimen without associated visual side effects [14]. Given the patient’s immunocompromised state, infections such as syphilis and toxoplasmosis could potentially lead to similar retinal findings. However, negative serology for these infections further reinforced the diagnosis of Stargardt disease.

## Conclusions

Stargardt disease is a hereditary retinal dystrophy caused by mutations in the ABCA4 gene, leading to progressive vision loss due to retinal atrophy. Human immunodeficiency virus/AIDS, characterized by severe immunosuppression, can exacerbate retinal degeneration through chronic inflammation, opportunistic infections, and potential effects of ART. This case illustrates the rare intersection of Stargardt disease and advanced HIV, highlighting the need for vigilant ophthalmologic monitoring and interdisciplinary care. It emphasizes the importance of understanding how systemic conditions like HIV can influence the progression of genetic retinal disorders, guiding tailored therapeutic approaches.
